# The Efficacy of Urinary Continence in Patients Undergoing Robot-Assisted Radical Prostatectomy with Bladder-Prostatic Muscle Reconstruction and Bladder Neck Eversion Anastomosis

**DOI:** 10.3390/medicina58121821

**Published:** 2022-12-10

**Authors:** Yang Luan, Xue-Fei Ding, Sheng-Ming Lu, Tian-Bao Huang, Ji Chen, Qin Xiao, Li-Ping Wang, Hao-Peng Chen, Yue-Xing Han

**Affiliations:** Clinical Medical College, Yangzhou University, Yangzhou 225009, China

**Keywords:** radical prostatectomy, urinary continence, muscle reconstruction, eversion, anastomosis

## Abstract

*Background and Objectives*: To evaluate the efficacy of bladder-prostatic muscle reconstruction and bladder neck eversion anastomosis in the recovery of urinary continence after robot-assisted radical prostatectomy (RARP). *Materials and Methods*: From January 2020 to May 2022, 69 patients who underwent RARP in our hospital were recruited. Thirty-seven patients underwent RARP with the Veil of Aphrodite technique (control group). On the basis of the control group, 32 patients underwent bladder-prostatic muscle reconstruction and bladder neck eversion anastomosis during RARP (observation group). The recovery of urinary continence was followed up at 24 h and 1, 4, 12, and 24 weeks after catheter removal. *Results*: There were no significant differences in operative time (127.76 ± 21.23 min vs. 118.85 ± 24.71 min), blood loss (118.27 ± 16.75 mL vs. 110.77 ± 19.63 mL), rate of leakage (3.13% vs. 2.70%), rate of positive surgical margin (6.25% vs. 10.81%), or postoperative Gleason score [7 (6–8) vs. 7 (7–8)] between the observation group and the control group (*p* > 0.05). After catheter removal, the rates of urinary continence at 24 h, 1 week, 4 weeks, 12 weeks, and 24 weeks were 46.88%, 68.75%, 84.38%, 90.63%, and 93.75% in the observation group, respectively. Meanwhile, the rates of urinary continence in the control group were 21.62%, 37.84%, 62.16%, 86.49%, and 91.89%, respectively. There was a significant difference between the two groups (*p* = 0.034), especially at 24 h, 1 week, and 4 weeks after catheter removal (*p* < 0.05). *Conclusions*: Bladder-prostatic muscle reconstruction and bladder neck eversion anastomosis were beneficial to the recovery of urinary continence after RARP, especially early urinary continence.

## 1. Background

In recent years, with the growth of the aging population and the popularization of early prostate cancer screening, the proportion of localized prostate cancer patients has increased [1,2]. For these patients, laparoscopic radical prostatectomy is one of the most important and effective treatment methods [3,4,5]. However, postoperative complications such as urinary incontinence and erectile dysfunction have seriously affected patients’ quality of life [6,7,8]. Compared with traditional laparoscopic radical prostatectomy, robot-assisted radical prostatectomy (RARP) has more advantages in postoperative tumour control, early urinary continence, and recovery of erectile function and benefits from its three-dimensional surgical field of view, flexibility in movement patterns, and the stability of the robotic arm [9,10,11]. In 2006, Savera et al. [12] first reported that the classic Veil of Aphrodite technique, which adopts the anterior approach of the bladder to the greatest extent possible to preserve the cavernous nerve, effectively preserves and protects the neurovascular bundle and achieves the ideal effect of early postoperative urinary continence. On the basis of mastering the Veil of the Aphrodite technique, we improved this procedure by reconstructing the bladder-prostate muscle and the eversion anastomosis of the bladder neck to the urethra. This paper retrospectively analysed the clinical data of 69 patients who underwent RARP using the improved method from January 2020 to May 2022 in our hospital and discussed the efficacy of the improved method.

## 2. Methods

### 2.1. Clinical Data

**Figure 1:**
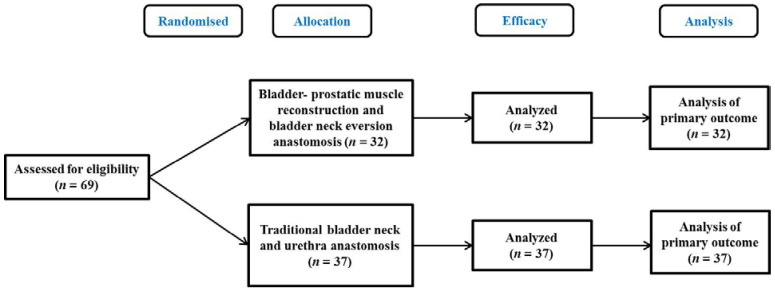
Flow diagram of the study.

We retrospectively analysed the clinical data of patients who underwent RARP in our hospital from January 2020 to May 2022. In the control group, 37 patients underwent RARP with the Veil of Aphrodite technique. On the basis of the control group, 32 patients in the observation group underwent improved RARP, which was bladder-prostatic muscle reconstruction combined with eversion anastomosis of the bladder neck to the urethra (Figure 1). There were no significant differences in age, BMI (body mass index), prostate volume, preoperative PSA (prostate specific antigen), or Gleason score between the two groups (*p* > 0. 05; Table 1). The study was approved by the ethics committee of the Clinical Medical College of Yangzhou University, and informed consents were obtained from the patients.

### 2.2. Surgical Technique

The same team of experienced surgeons performed all operations. The surgery was performed with a standard Da Vinci Surgical Robot (Intuitive Surgical, USA) and used the traditional W-type five casing. The operation was performed via an extraperitoneal approach and an anterior approach.

The Veil of Aphrodite technique: 1. Separate the posterior pubic space; clean the fat on the anterior surface of the prostate and the connective tissue on the surface of the intra-pelvic fascia; open the intra-pelvic fascia; separate the prostatic pubic ligament; and suture the deep dorsal vein complex. 2. Drag the catheter, identify the junction between the prostate and the bladder neck, and separate the bladder neck. 3. Separate the vas deferens and seminal vesicles, cut off both sides of the vas deferens, transversally cut the Denonvillier fascia, expose the posterior space of the prostate, retain the neurovascular bundles on both sides, and dissociate the posterior part of the prostate from the apex of the prostate. 4. The anterior wall of the urethra was cut close to the tip of the prostate, and the lateral wall and posterior wall of the urethra were exposed and cut off. The rectourethral muscle near the tip of the prostate was cut off, and the prostate was completely removed.

**Figure 2:**
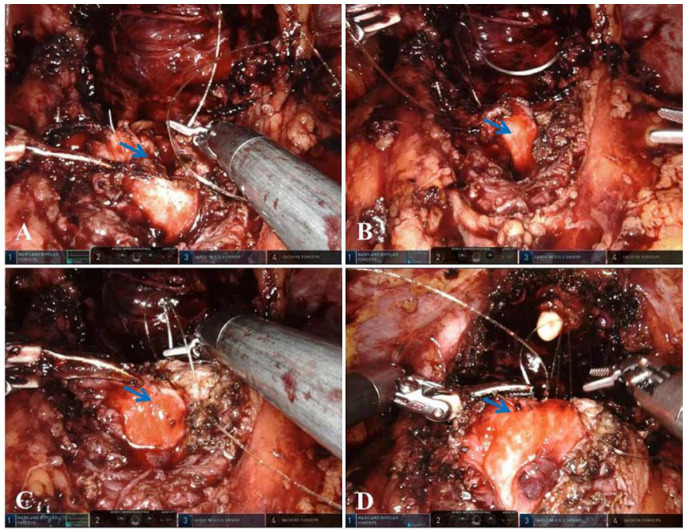
The important steps of bladder-prostatic muscle reconstruction and eversion anastomosis of the raised bladder neck in robot-assisted radical prostatectomy. (**A**), Severed bladder-prostatic muscle was transversely anastomosed behind the bladder neck to reconstruct and strengthen it (bladder-prostatic muscle: blue arrow); (**B**), A longitudinal “ridge” formed in the bladder neck after the bladder-prostatic muscle was transversely anastomosed (ridge: blue arrow); (**C**,**D**), The bladder neck was everted, raised, and anastomosed with the urethra (blue arrow: eversion and elevation of the posterior wall of the bladder neck).

Bladder-prostatic muscle reconstruction: The severed bladder-prostatic muscle was transversely anastomosed behind the bladder neck to reconstruct and strengthen it (Figure 2A), while a longitudinal “ridge” formed in the bladder neck (Figure 2B).

Bladder neck eversion: Identify and protect the bilateral ureteral openings; anastomose the bladder neck at 6 o’clock with the midline of the posterior wall of the bladder approximately 1 cm away from the bladder neck (Figure 2C). The above longitudinal anastomosis was performed at 5 o’clock and 7 o’clock of the bladder neck with the corresponding posterior wall of the bladder successively, which can fold the posterior wall of the bladder and elevate the bladder neck. Finally, the bladder neck was anastomosed with the stump of the urethra (Figure 2D).

The operative time, estimated blood loss, rate of leakage, rate of positive surgical margins, and postoperative pathology were recorded in the two groups. In all patients, the catheter was removed at two weeks postoperatively. We followed up on the number of pads used at 24 h (immediate continence) and 1, 4, 12, and 24 weeks after catheter removal.

Postoperative urinary continence criteria: the use of 0-1 pads per day was defined as continence, and the use of ≥2 pads per day was defined as urinary incontinence.

## 3. Statistical Analysis

The SPSS 19.0 statistical software was used to process the data. Measurement data are represented as $\bar{x}$ ± s, and the Student’s t test was used for comparisons between groups. Grade data are displayed as the median (interquartile range), and the Mann-Whitney U test was used for comparisons between groups. Count data are expressed as [example (%)], and the x^2^ test was used. *p* < 0.05 indicates that the difference is statistically significant.

## 4. Results

Operations in both groups were successfully completed, and there was no transfer to open surgery or blood transfusion. The operative time and intraoperative blood loss in the observation group were 127.76 ± 21.23 min and 118.27 ± 16.75 mL, respectively. The operative time and intraoperative blood loss in the control group were 118.85 ± 24.71 min and 110.77 ± 19.63 mL, respectively. There was no significant difference between the two groups (*p* > 0.5). One patient in both the observation group and the control group had anastomotic leakage after RARP, and there was no significant difference between the two groups (*p* = 0.917).

There were two patients (6.25%) with positive margins in the observation group and four patients (10.81%) with positive margins in the control group, with no statistical significance (*p* = 0.498). The postoperative Gleason scores of the observation group and the control group were 7 (6–8) and 7 (7–8), respectively. There was also no significant difference between the two groups (*p* = 0.943) (Table 2).

The catheter was removed two weeks after surgery in both groups. In the observation group, the rates of urinary continence at 24 h, 1 week, 4 weeks, 12 weeks and 24 weeks after catheter removal were 46.88% (15/32), 68.75% (22/32), 84.38% (27/32), 90.63% (29/32) and 93.75% (30/32), respectively. Meanwhile, urinary continence rates in the control group at 24 h, 1 week, 4 weeks, 12 weeks and 24 weeks after catheter removal were 21.62% (8/37), 37.84% (14/37), 62.16% (23/37), 86.49% (32/37) and 91.89% (34/37), respectively. There was a significant difference between the two groups (*p* = 0.034), especially at 24 h, 1 week, and 4 weeks after catheter removal, and the rates of urinary continence in the observation group were significantly better than those in the control group (*p* < 0.05) (Table 2).

## 5. Discussion

Complications, such as urinary incontinence, often occur after radical prostatectomy, which has a great effect on patients’ quality of life [13,14,15]. In addition, among the three important evaluation indices proposed by Salomon et al. [16], including tumour control, urinary continence and sexual function recovery, postoperative urinary continence has always been an important criteria for evaluating the efficacy of radical prostatectomy. Since the advent of robot-assisted surgery systems, RARP has gradually become the standard surgical method for the treatment of localised prostate cancer and has been successively carried out and popularised in many centres worldwide [11,17]. In addition to its inherent advantages, robot technology has also enhanced surgeons’ understanding of prostate anatomy. Scholars around the world continue to try and exploit new technologies to speed up the early recovery of postoperative urinary continence. 

At present, there are two kinds of technology: the anterior approach and the posterior approach, which are more popular and recognized. The anterior approach and the posterior approach are represented by the Veil of the Aphrodite technique [12] and the Retzius-sparing technique [18], respectively. The Veil of the Aphrodite technique, which first opens the retzius, exposes the anterior bladder wall, retains the lateral prostate capsule, and dissects from interfascial to intrafascial anatomical to maximise the retention of the cavernous nerve, is a classic RARP for nerve preservation. This procedure can not only preserve and protect the neurovascular bundle well, but it also has many advantages, such as a large operating space, obvious anatomical signs, clear anatomical structure in the operative field, and convenient anastomosis of the urethra and bladder neck. In 2010, Galfano et al. [19] first proposed the preservation of the retropubic space approach. Different from the anterior approach, the posterior approach does not need to open the retropubic space and can better preserve the structures in front of the prostate related to urinary continence, such as the dorsal vein complex, puboprostatic ligament, intra-pelvic fascia, and accessory pudendal artery [20,21]. However, the posterior approach also has some disadvantages: more limited operating space, a greater difficulty coefficient, a longer learning curve, more intraoperative bleeding, easier injury to the bladder wall and ureter, etc. [22]; Since the perspective and sequence of cysto-urethral anastomosis in this operation are different from those in the anterior approach and it is performed underneath the bladder, it is more difficult [19]. For anterior and late-stage prostate cancer, the posterior approach is not recommended due to positive surgical margins [22,23]. Therefore, on the basis of mastering the Veil of Aphrodite technology, this study intends to improve it to accelerate the recovery of early urinary continence after RARP without increasing the difficulty of the operation and ensuring safety.

Studies have shown that preserving the bladder neck during RARP is helpful for the recovery of early urinary continence [24,25,26]. However, when the prostate is separated from the bladder neck, the bladder-prostatic muscle will inevitably be injured. At the posterior of the bladder neck, the bladder-prostatic muscle connects the bladder to the prostate, and studies have shown that the muscle plays an important role in controlling urination, and its reconstruction can increase muscle strength and accelerate the recovery of urinary continence [27,28]. In addition, after anastomosis of the bladder and urethra, the urination pressure can be directly transferred from the bladder cavity to the urethra, which is not conducive to urinary continence. 

Hence, in this study, we reconstructed the bladder-prostatic muscle to increase its strength and form a “ridge” on the posterior wall of the bladder neck that protrudes into the bladder. Then the posterior wall of the bladder neck was everted to raise the bladder neck. The advantages of eversion and elevation of the bladder neck and anastomosis with the urethra are as follows: On the one hand, it can elevate and narrow the bladder neck, change the urination angle, buffer the urination pressure, and increase the urethral closure pressure; on the other hand, it can effectively reduce the formation of anastomotic scars, help relieve urethral stricture, and promote the recovery of early urinary continence. Our study showed that, compared with the traditional anterior approach to RARP, the improved anterior approach to RARP did not increase the operative time, postoperative blood loss, or rate of postoperative urine leakage. Moreover, the improved anterior approach to RARP achieved similar tumour control results as the traditional anterior approach to RARP. However, in terms of urinary continence, our study showed that the improved anterior approach to RARP was significantly better than the traditional anterior approach to RARP, especially in the immediate and early recovery of urinary continence. The efficacy of urinary continence was even comparable to or even better than that of RARP through the posterior approach [29,30,31]. 

Some limitations of the present study should be acknowledged. Due to the small sample size, short postoperative follow-up time, and strong subjective efficacy of this study, further verification of long-term prospective studies with large samples is needed. No postoperative urography or cystoscopy was performed to further clarify the changes in the urination angle. The improved anterior approach achieved a better urinary continence rate without increasing the difficulty of the operation, but the surgical technique was still high, and it needed to be carried out by physicians with certain experience in robotic surgery. Moreover, we further collected the membranous urethra length, intravesical prostate midlobe, apex morphology, and other factors to explore whether they affect the recovery of urinary continence, with a view to further improving the quality of life of patients.

In conclusion, reconstruction of the bladder-prostatic muscle and eversion anastomosis of the raised bladder neck with the urethra play an important role in improving the recovery of early urinary continence after RARP. At the same time, this method ensures tumour control efficacy and does not increase the complications and complexity of the operation. It is a feasible and effective surgical technique.

## Figures and Tables

**Table 1:** Participants’ characteristics.

Characteristics	Observation Group	Control Group	*p*-Value
Age (Years)	67.53 ± 4.76	65.72 ± 6.31	0.189
BMI (kg/m^2^)	24.15 ± 3.21	25.17 ± 3.44	0.210
Prostate volume (mL)	40.19 ± 14.15	38.54 ± 16.36	0.658
PSA(ng/mL)	17.12 ± 12.37	19.14 ± 9.69	0.450
Gleason score [*n* (%)]			0.893
6	8 (25.00)	9 (24.32)	
7	15 (46.88)	20 (54.05)	
8	6 (18.75)	6 (16.21)	
≥9	3 (9.38)	2 (5.41)	

BMI = body mass index; PSA = prostate-specific antigen.

**Table 2:** Perioperative and histopathologic data in two groups.

Groups	Observation Group	Control Group	*p*-Value
Operative time (min)	127.76 ± 21.23	118.85 ± 24.71	0.116
Blood loss (mL)	118.27 ± 16.75	110.77 ± 19.63	0.095
Rate of leakage (%)	3.13 (1/32)	2.70 (1/37)	0.917
Rate of positive surgical margin (%)	6.25 (2/32)	10.81 (4/37)	0.498
Postoperative Gleason score [*n* (%)]			0.943
6	7 (21.88)	8 (21.62)	
7	14 (43.75)	17 (45.95)	
8	7 (21.88)	9 (24.32)	
≥9	4 (12.50)	3 (8.11)	
Urinary continence rates [*n* (%)]			0.034
24 h	15 (46.88)	8 (21.62)	0.026
1 week	22 (68.75)	14 (37.84)	0.010
4 weeks	27 (84.38)	23 (62.16)	0.039
12 weeks	29 (90.63)	32 (86.49)	0.874
24 weeks	30 (93.75)	34 (91.89)	1.000

## Data Availability

The data presented in this study are available on request from the corresponding author. The data are not publicly available due to privacy or ethical restrictions.
